# County Sanitary Associations and Their Uses1Read at the forty-first annual meeting of the Medical Association of Central New York, held at Buffalo, October 27, 1908.

**Published:** 1909-09

**Authors:** O. J. Hallenbeck

**Affiliations:** Canandaigua, N. Y.


### County Sanitary Associations and Their Uses[1]

BY O. J. HALLENBECK, M. D., Canandaigua, N. Y.

AT first thought, it may seem out of place to read a paper of this nature at such a gathering as this. The title suggests a paper to be read only to a gathering of health officers at some sanitary meeting. That sanitation may be effectual, it requires the united coöperation of every physician, be he a general or a special practitioner. The people appeal to the physician to learn how to mitigate the spread of diseases, and to enlighten them as to those environments and conditions, which will best preserve their health. This is the age of preventive medical practice, and the practitioner who most successfully prevents the spread of infectious and contagious diseases, is a most valuable asset to the community in which he practices. Report at once all cases of infectious and contagious diseases to your health officer, and if you are in doubt as to the exact nature of the disease, give the community the benefit of the doubt by an immediate report.

If the health officer is alert and responds to his duties as the community have a right to expect he should, many a serious calamity will be averted. Should he be incompetent or neglectful of his duties, let him step aside or if needs be, the citizens should demand that he step down and out, in order that another may direct and supervise those matters that are so vital both to the sick, and to those who must necessarily come in contact with the afflicted. It is often said that health officers are poorly paid, but you and I have seen towns, villages and cities pay good money for very poor services rendered. One object of this paper is to impress practitioners with the fact that they ought to support the health officer, for without their support and encouragement, his endeavors however meritorious will not prove successful. The other is to stimulate health officers to efficiency, and to make them feel that they have taken upon themselves a great responsibility, when they assume the office of supervisor of the public health.

---

1. Read at the forty-first annual meeting of the Medical Association of Central New York, held at Buffalo, October 27, 1908.

It requires no argument to convince those present that being a member of the Medical Society of the State of New York, is all the medical organisation desirable and beneficial for any medical practitioner. Without being a member of, and attending the meetings of one or more of the local, county, district, or state societies, one soon gets bound up in a mold of selfishness, narrowness, and in a self-satisfying condition, a menace to himself and a greater menace to the people whom he serves. For a health officer to be a member of, and attend the annual meeting of the State Sanitary Association does not suffice to inhibit stimulation for twelve months. The demand for efficient health officers in the towns and villages of the state, are of as much importance as in the cities, even more so, for cities are more proficient in sanitary matters. I had been of the opinion for some time, that sanitary supervision of towns, villages, and small cities of the state, would be much more efficient if each county had its own sanitary association. Having counseled with several physicians, some of whom were health officers, in regard to the benefits to be derived from such an organisation, sufficient encouragement was received to warrant the undertaking.

A letter setting forth the object of the meeting was sent to each health officer in the county to meet in Canandaigua, March 4, 1906, for the purpose of organising a County Sanitary Association. Ontario County has twenty-one health officers, and fourteen were present at the appointed meeting, as were also several physicians from the village

Ways and means of organising were discussed, and a committee of three was appointed to draft a constitution and by-laws for its government, the same to report at an adjourned meeting to be called by the president. The adjourned meeting was held October 30, 1906, at which time a permanent organisation was effected, and a constitution and by-laws were adopted. Among other things they provide that each and every health officer of the county is an active member of the association, and every physician of the county, may become an honorary member. The annual dues to the association shall be one dollar for each Board of Health of the county, and payable by them through their health officer. Each specimen to be analysed or examined by the county bacteriologist, must be validated by a duly qualified physician or health officer.

The executive committee shall have general supervision over the bacteriologist, and the Ontario County laboratory. It shall recommend to the Board of Supervisors of Ontario County, at their annual meeting a bacteriologist for their appointment. It shall inspect the laboratory from time to time, and acquaint itself as to the quality and quantity of work done, and judge as to the competency or incompetency of the bacteriologist; it shall recommend his removal if it is found that he is not competent and successful in the work for which he is employed; it shall require him to make a detailed report both to the board of supervisors and to the executive committee quarterly; it shall from time to time consult with the advisory committee of the Ontario County Medical Society, with the Society of Physicians and Surgeons of the Village of Canandaigua, and the Geneva Medical Society, in regard to matters pertaining to the laboratory and its work.

In order that a county laboratory shall fulfil the mission for which it shall have been created, it must be supervised by the medical men of the county. They in turn should be responsible to the board of supervisors, who represent its citizens and who are the beneficiaries, and bear the burden of expense. Sanitary officers are educators and directors of the needs of the citizens in sanitary matters, particularly when infectious and contagious diseases prevail. When organised their influence is extended and their power is felt to the remotest districts of the county. I am convinced, from our short experience as a county sanitary association, that contagious and infectious diseases are receiving that attention and care which did not prevail prior to our organisation. Now each health officer may know what is being done by every other one. and he is encouraged to do his best, knowing that his success or failure will be known by his neighbor. At present we are making a canvass to locate every case of tuberculosis and cancer in our county. Printed slips have been supplied to every physician in the county, on which a brief history of each case is recorded, and then filed with the health officer of the district in which he resides. If each health officer and physician performs his duty, as we have a right to expect he will, the executive committee at the next quarterly meeting, should know the number, location, and condition of every case of tuberculosis in the county. This data having been obtained, we can go before the board of supervisors with definite facts, and recommend whatever seems best to us. not only from a financial, but also from a humanitarian point of view.

